# Absolute and relative GFR and contrast medium dose/GFR ratio: cornerstones when predicting the risk of acute kidney injury

**DOI:** 10.1007/s00330-023-09962-w

**Published:** 2023-08-04

**Authors:** Ulf Nyman, Peter Leander, Per Liss, Gunnar Sterner, Torkel Brismar

**Affiliations:** 1 Department of Translational Medicine, Division of Medical Radiology, University of Lund, Malmö, Sweden; 2 Department of Surgical Sciences, Section of Radiology, Uppsala University, Uppsala, Sweden; 3 Department of Nephrology, Skåne University Hospital, Malmö, Sweden; 4 Division of Medical Imaging and Technology, Department of Clinical Science, Intervention and Technology (CLINTEC), Karolinska Institute/Karolinska University Hospital, Stockholm, Sweden; 5 Department of Radiology, Karolinska University Hospital in Huddinge, Stockholm, Sweden

**Keywords:** Acute kidney injury, Angiography, Computed tomography, Contrast media, Glomerular filtration rate

## Abstract

**Abstract:**

Glomerular filtration rate (GFR) is considered the best overall index of kidney function in health and disease and its use is recommended to evaluate the risk of iodine contrast medium-induced acute kidney injury (CI-AKI) either as a single parameter or as a ratio between the total contrast medium dose (gram iodine) and GFR. GFR may be expressed in absolute terms (mL/min) or adjusted/indexed to body surface area, relative GFR (mL/min/1.73 m^2^). Absolute and relative GFR have been used interchangeably to evaluate the risk of CI-AKI, which may be confusing and a potential source of errors. Relative GFR should be used to assess the GFR category of renal function as a sign of the degree of kidney damage and sensitivity for CI-AKI. Absolute GFR represents the excretion capacity of the individual and may be used to calculate the gram-iodine/absolute GFR ratio, an index of systemic drug exposure (amount of contrast medium in the body) that relates to toxicity. It has been found to be an independent predictor of AKI following percutaneous coronary angiography and interventions but has not yet been fully validated for computed tomography (CT). Prospective studies are warranted to evaluate the optimal gram-iodine/absolute GFR ratio to predict AKI at various stages of renal function at CT. Only GFR estimation (eGFR) equations based on standardized creatinine and/or cystatin C assays should be used. eGFR_cystatin C_/eGFR_creatinine_ ratio < 0.6 indicating selective glomerular hypofiltration syndrome may have a stronger predictive power for postcontrast AKI than creatinine‐based eGFR.

**Clinical relevance statement:**

Once the degree of kidney damage is established by estimating *relative* GFR (mL/min/1.73 m^2^), contrast dose in relation to renal excretion capacity [gram-iodine/*absolute* GFR (mL/min)] may be the best index to evaluate the risk of contrast-induced kidney injury.

**Key Points:**

• *Relative glomerular filtration rate (GFR; mL/min/1.73 m*^*2*^*) should be used to assess the GFR category as a sign of the degree of kidney damage and sensitivity to contrast medium-induced acute kidney injury (CI-AKI).*

• *Absolute GFR (mL/min) is the individual’s actual excretion capacity and the contrast-dose/absolute GFR ratio is a measure of systemic exposure (amount of contrast medium in the body), relates to toxicity and should be expressed in gram-iodine/absolute GFR (mL/min).*

• *Prospective studies are warranted to evaluate the optimal contrast medium dose/GFR ratio predicting the risk of CI-AKI at CT and intra-arterial examinations.*

**Supplementary Information:**

The online version contains supplementary material available at 10.1007/s00330-023-09962-w.

## Introduction

GFR (glomerular filtration rate) is considered the best overall index of kidney function in health and disease [1]. In a radiology context, its use is recommended to evaluate the risk of iodine contrast medium-induced acute kidney injury (CI-AKI) [2–4], either as a single parameter or as a ratio between the total contrast medium (CM) dose and GFR [3, 5]. The CM dose/GFR ratio is a pharmacokinetic index for systemic drug exposure [6, 7] and has been validated for the estimation of CI-AKI risk at percutaneous coronary angiography (PCA) and interventions (PCI) [8–10] but not yet for computed tomography (CT). Since the actual risk of CI-AKI at CT still remains uncertain in patients with moderate-severe and severe kidney disease and without considering individual CM doses [2, 11], a more accurate risk prediction may be obtained if combining the two basic risk factors, CM dose and renal function, into one variable.

GFR may be measured (mGFR) indirectly by analyzing plasma or renal clearance of externally injected biomarkers [12, 13], but is not practical to use in a busy daily radiology practice. Instead, GFR may be estimated (eGFR) using equations based on plasma/serum creatinine, cystatin C or both [1, 14]. Both mGFR and eGFR may be expressed in absolute terms, mL/min, or adjusted to body surface area (BSA), relative GFR in mL/min/1.73 m^2^. Absolute and relative GFR have been used interchangeably to evaluate the risk of CI-AKI, both in terms of the GFR value itself [3, 5] and CM-dose/GFR ratio [8, 10]. The lack of a clear distinction when to use absolute and relative GFR may be confusing and a potential source for errors. European Medicines Agency, US Food and Drug Administration, and Kidney Disease: Improving Global Outcomes (KDIGO) states that the dosing of drugs excreted by glomerular filtration should be based on absolute GFR, i.e., without adjustment/indexation for BSA [15–17]. However, this is only partly correct. With regard to nephrotoxic CM, the patient’s GFR adjusted to BSA (relative GFR) should also be evaluated to establish the level of renal function as a sign of possible renal damage [18] and a measure of nephrotoxic sensitivity of the present agent.

The purpose of the present paper is to discuss and clarify the role of absolute and relative GFR when evaluating the risk of CI-AKI, if experiences of CM dose/GFR ratio from PCA/PCI may also be used for CT and to encourage the use of dose/GFR ratio in CI-AKI research of CM-enhanced CT. We also report on a new way of evaluating CI-AKI risk using the eGFR_cystatin C_/eGFR_creatinine_ ratio.

## Glomerular filtration rate

### Absolute GFR (mL/min)

**Fig. 1:**
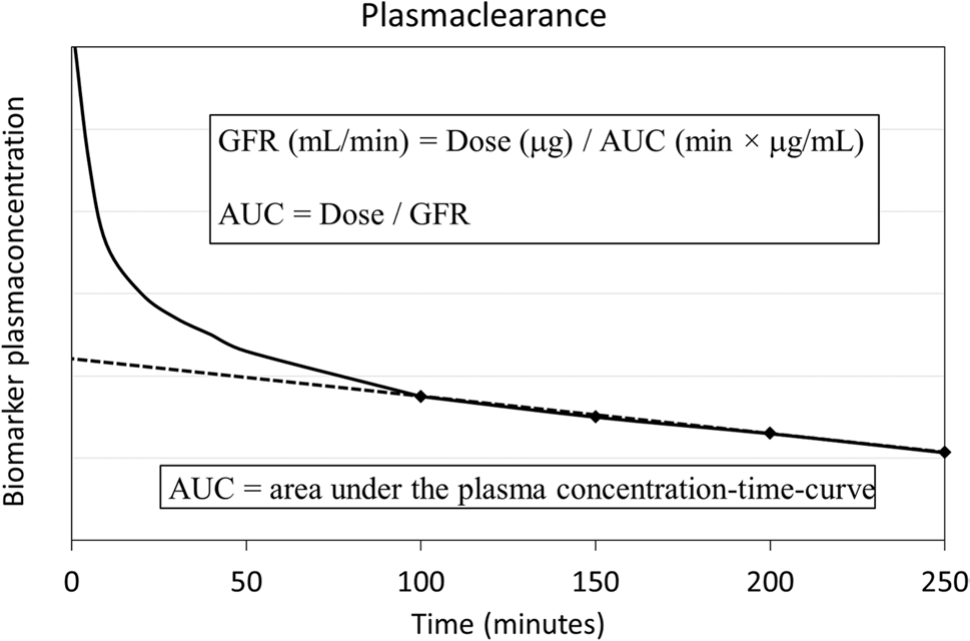
Plasma clearance of an externally injected biomarker such as iohexol at time zero based on the plasma concentration curve (solid line) as a function of time. The plasma concentration rapidly reaches a peak followed by an exponential fall as the biomarker is diluted in circulating plasma, diffuses into the interstitial space (volume distribution), and reaches an equilibrium after about 2 h at normal renal function. Thereafter follows the elimination phase where the plasma concentration of the biomarker falls at a constant rate. According to the one-compartment model, the *area under the plasma concentration time-curve* (AUC, dashed line) can be calculated based on four plasma samples (diamond points) obtained 100–250 min after the bolus injection. The absolute glomerular filtration rate (GFR_ABS_, mL/min) can be calculated by injected dose (μg) / AUC (min × μg/mL) from which follows that AUC = dose/GFR_ABS_, a measure of systemic exposure of the injected agent

Plasma clearance of an injected filtration marker such as iohexol is illustrated in Fig. 1. It illustrates a one-compartment model where the plasma concentration of the marker is obtained at four time points when equilibrium has been achieved after the initial redistribution into the interstitial space followed by the excretion phase [19]. GFR may then be calculated accordingly:

1 $$\mathrm{GFR}\;(\mathrm{mL}/\min)=\mathrm{Injected}\;\mathrm{dose}(\mathrm{\mu g})/\mathrm{AUC}\;(\min\times\mathrm{\mu g}/\mathrm{mL})$$

where AUC denotes the area under the plasma concentration–time curve.

The calculated measure describes GFR in absolute terms (GFR_ABS_, mL/min), i.e., regardless of body size, and represents the *excretion capacity* of the individual. Since absolute GFR (GFR_ABS_) equals injected dose/AUC, then AUC equals injected dose/GFR_ABS_.

### AUC = systemic exposure

According to the pharmacokinetics of drugs that are distributed and eliminated according to linear kinetics such as contrast media, *AUC* represents a measure of systemic exposure [6–8]. From Eq. 1, it can be deduced that AUC is equal to injected dose/GFR_ABS_. Systemic exposure is often well correlated with the toxicity of a drug and hence generally used as an index for dose optimization. Thus, the toxicity of a drug is related to the total injected dose and the excretion capacity (absolute GFR) of the individual. The higher the injected dose and the lower the excretion capacity, the longer time is required until the elimination of the offending drug and the higher the risk of side effects. When it comes to CM, this relation may be summarized as *gram-iodine/GFR ratio based on absolute GFR* [7, 20].

### Relative GFR (mL/min/1.73 m.2)

Renal function is proportional to body size. To establish normal reference intervals and categories of decreased kidney function and damage, GFR must be adjusted/indexed to a certain body size, commonly denominated relative GFR [18]. By tradition 1.73 m^3^ body surface area (BSA) has been used for indexing, the mean BSA value for 25-year-old men and women in the USA in the 1920s [21]. Thus, after obtaining a measured absolute GFR it is indexed to 1.73 m^2^ by the following calculation:

2 $$Relative\;GFR\;(\mathrm{mL/min/1.73\ m^2)}=(\mathrm{absolute}\;\mathrm{GFR}/\mathrm{BSA})\times1.73$$

Notably modern creatinine-based GFR estimating equations such as MDRD, CKD-EPI, EKFC, and LMR [22, 23] primarily estimate relative GFR*.* To obtain absolute GFR values deindexation must be performed according to:

3 $$Absolute\;GFR\;(\mathrm{mL}/\min)=(\mathrm{relative}\;\mathrm{GFR}/1.73)\;\times\;\mathrm{BSA}$$

According to the Civilian American and European Surface Anthropometry Resource project published in 2001, the mean BSA for women and men is 1.73 m^2^ and 2.03 m^2^, respectively [24]. However, the indexation of GFR to BSA is not without criticism and may be misleading in certain populations, such as those with body mass index < 18.5 or > 30 kg/m^2^ [21, 25].

The most commonly used formula for determining BSA still today is the one presented by Du Bois and Du Bois 1916 [26]:

4 $$BSA = {\mathrm{weight}}^{0.425} (\mathrm{kg}) \times {\mathrm{height }(\mathrm{cm})}^{0.725} \times 0.007184$$

Though a number of formulas have been developed since 1916, their superiority to the Du Bois formula remains to be clearly proven [21, 24].

Chemical laboratories most commonly report GFR in mL/min/1.73 m^2^ and it is the unit used to classify GFR categories as a sign of possible kidney damage (Table 1). The classification based on relative GFR should primarily be used when evaluating the risk of CI-AKI. Depending on the GFR category, the vulnerability of the kidneys may then vary with the amount of CM circulating in the body, i.e., systemic exposure. As mentioned above, systemic exposure can be described by the gram-iodine dose/GFR_ABS_ ratio. Table 2 illustrates how relative GFR and systemic exposure may vary with body size despite the same absolute GFR.

**Table 1:** GFR categories as a sign of kidney damage according to KDIGO 2013 (Kidney Disease: Improving Global Outcomes) [18]

GFR categories (mL/min/1.73 m^2^)		Renal function
G1	≥ 90	Normal or high
G2	60–89	Mildly decreased
G3a	45–59	Mildly to moderately decreased
G3b	30–44	Moderately to severely decreased
G4	15–29	Severely decreased
G5	< 15	Kidney failure

*GFR* glomerular filtration rate

**Table 2:** Indexed renal function and systemic exposure of CM for abdominal CT in individuals with increasing body size, but with the same renal excretion capacity. Note that an excretion capacity of 45 mL/min (absolute GFR) indicates only a mildly decreased renal function (relative GFR 75 mL/min/1.73 m^2^) in a small individual (130 cm/30 kg) but moderately-severely damaged kidneys (relative GFR 36 mL/min/1.73 m^2^) in a large individual (190 cm/90 kg). When providing the same dose of CM per kg body weight there is a low g-I/GFR ratio (0.33) for the small individual and an anticipated minimal risk of CI-AKI while for the large individual, it results in a relatively high ratio (1.00) and thus a greater CI-AKI risk

Body size			Renal excretion capacity	Indexed renal function	Computed tomography	Systemic exposure
Height (cm)	Weight (kg)	BSA (m^2^)	Absolute GFR (mL/min)	Relative GFR (mL/min/1.73 m^2^)	CM-dose (mg-I/kg)	g-I/GFR_ABS_ ratio
130	30	1.04	45	75	500	0.33
140	40	1.24	45	63	500	0.44
150	50	1.43	45	54	500	0.56
*170*	*63*	*1.73*	*45*	*45*	500	0.70
180	80	2.00	45	39	500	0.89
190	90	2.18	45	36	500	1.00
200	100	2.37	45	33	500	1.11

*ABS* absolute, *BSA* body surface area, *CI-AKI* contrast medium-induced acute kidney injury, *CM* contrast medium, *GFR* glomerular filtration rate, *mg/g-I* milligram/gram iodine

Notably, equations to estimate GFR are only meaningful to use in patients with a steady state renal function. Therefore, they will perform less accurately in acute conditions with unstable renal function, e.g., cardiac failure or hypotension, since it will take time for both cystatin C and especially creatinine to reach a new steady state plasma level [27].

## Contrast medium dose/GFR ratio

Mounting evidence from PCA/PCI studies (Table S1 and S2) [8, 9, 28–39] indicate that a ratio between CM-volume (mL) or gram-iodine (g-I) and estimated GFR represents a significant and independent predictor of post-contrast medium AKI (PC-AKI), first suggested by Altmann et al 1997 [40]. Though the CM dose/GFR ratio indicating an increased risk of PC-AKI may vary substantially between studies, from a g-I/GFR ratio of about 0.5 to 2.0. The weighted mean cut-off value to predict an increased risk of PC-AKI was 1.0 for the entire group, 1.24 for studies based on absolute GFR with the Cockcroft-Gault (CG) equation, and 0.77 for studies based on relative GFR with the MDRD and CKD-EPI equations (Table S2). The variation among the studies may be the result of differences in e.g. study design, indication, patient selection, and variation in comorbidities.

**Fig. 2:**
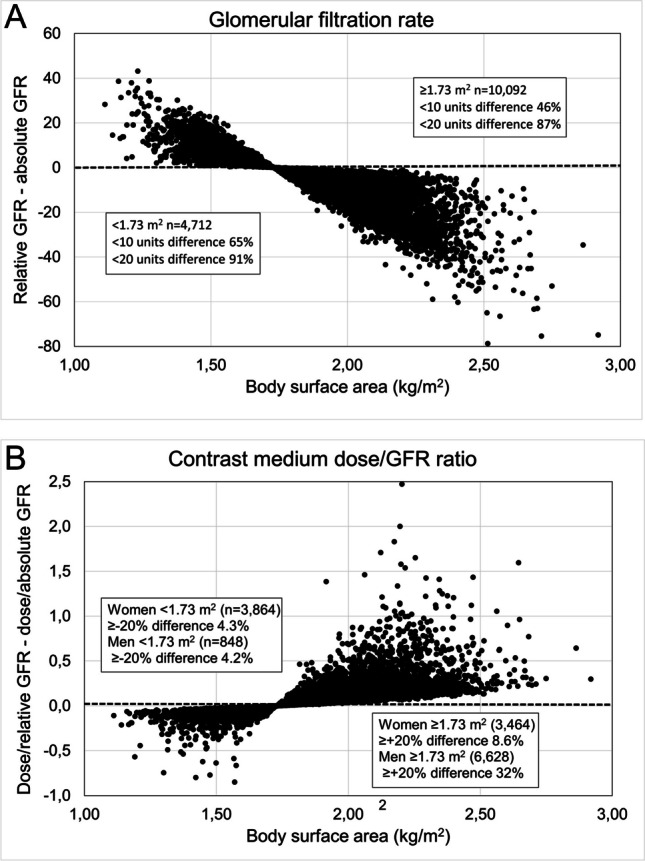
Diagrams to illustrate the differences between (**A**) relative GFR (mL/min/1.73 m^2^) and absolute GFR (mL/min) and (**B**) gram-iodine/GFR ratio based on relative and absolute GFR in relation to body surface area. A simulated CT contrast medium dose of 500 mg I/mL was applied to a cohort of adult patients in reference [41] with a wide range of GFR and body surface area. The original study [41] concerned the performance of creatinine-based GFR estimating equations in the context of drug dosage adjustment. Mean/median difference between relative and absolute GFR -4/-5 units. Median body surface area women 1.72 m^2^ (*n* = 7328) and men 1.96 m.^2^ (*n* = 7476)

To merge data from studies using absolute and relative GFR to establish a weighted cut-off dose/GFR ratio to predict PC-AKI may be questioned. However, a comparison of the area under the receiver-operator characteristic curve demonstrated no difference in predicting AKI between CM-dose/absolute GFR based on the CG equation and CM-dose/relative GFR based on the 186-MDRD equation [10]. This may be explained by the fact that though the difference between relative and absolute GFR varies with BSA, the difference was relatively small in the majority of patients and with an average difference of only about five units in a Western population with a median BSA of 1.84 kg/m^2^ (Fig. 2A). In three of the Asian CM-dose/GFR ratio studies, BSA was reported or calculated by us to be 1.67–1.70 kg/m^2^ [36, 38, 39] indicating an even smaller average difference between absolute and relative GFR. Still, when applying the ratio to small and large individuals to predict the risk of CI-AKI, significant ratio differences may occur depending on whether relative or absolute GFR (Fig. 2B). The ratio should express the amount of potentially toxic CM (systemic exposure) relative to injected dose and individual kidney function. This is not reflected by relative GFR since it is adjusted to a fixed body size, 1.73 m^2^ BSA. Only absolute GFR gives the true excretion capacity of the individual.

Among the studies, there was only one large retrospective registry study (*n* = 2306) observing a high ratio threshold (2.15 g-I/GFR ratio) [29], while two other large retrospective registry studies (*n* > 1000) [8, 33], one large prospective single-center study (*n* = 3273) [31] and one large multicenter study (*n* = 4254) [37] all reported lower thresholds, ranging from 0.62 to 1.30. When applying the observed threshold of 0.62 on subsets of patients with different risk factors in the multicenter study by Nie et al [37], the incidence of PC-AKI in almost all subsets was about 4 times higher in those with a ratio above the threshold (Table 3). In a study on hydration, Liu et al [31] found a higher sensitivity to the g-I/GFR ratio in dehydrated patients with an optimal threshold of 0.69 at insufficient hydration, but of 1.08 in those who were sufficiently hydrated.

**Table 3:** Incidence of acute kidney injury (serum creatinine rise ≥ 0.5 mg/dL) below and above the optimal gram-iodine/GFR ratio (using relative GFR) to predict acute kidney injury in subsets of patients undergoing percutaneous coronary angiography or interventions (PCI) according to Fig. 3 in reference [37]. Regarding Mehran score see reference [42]

	Incidence of acute kidney injury (%)		*p* values
Gram-iodine/GFR_REL_ ratio	< 0.62*	≥ 0.62*	
Age < 60	0.5	2.7	< 0.001
Age ≥ 60	1.1	4.3	< 0.001
Elective PCI	0.7	3.6	0.029
Emergent PCI	1.8	4.6	< 0.001
No STEMI	0.8	3.2	< 0.001
STEMI	1.0	5.6	< 0.001
No cardiogenic shock	0.6	2.9	< 0.001
Cardiogenic shock	*3.5*	*10.6*	*0.709*
LVEF ≥ 40%	0.7	4.9	< 0.001
LVEF < 40%	3.9	14.1	0.010
Low Mehran score	*0.5*	*0.8*	*0.322*
High Mehran score	1.7	6.2	0.0015

^*^Based on a CM-volume/GFR ratio < 1.78 and anticipating a concentration of 350 mg I/mL

Italic = non-significant; *CM* contrast medium, *LVEF* left ventricular ejection fraction, *STEMI* ST-elevated myocardial infarction

In the Blue Cross Blue Shield of Michigan Cardiovascular Consortium registry study [10], not included in Table S1 and S2, evaluating the risk of PC-AKI and the need for in-hospital dialysis in 58,957 patients undergoing PCI, it was recommended to restrict the CM-volume (concentration not specified) to less than thrice and preferably less than twice the GFR-value. That corresponds to a g-I/GFR ratio of 1.05 and 0.70, respectively, anticipating a mean CM concentration of 350 mg I/mL during PCI. Follow-up studies from the same registry showed that the use of high CM doses (g-I/GFR ratio > 1.0) at the time of PCI was associated with increased risk of PC-AKI and need for dialysis, regardless of their baseline predicted risk of these complications [43]. Post hoc analyses have later estimated that an across-the-board 30% reduction of CM dose would be expected to prevent one-eighth of the AKI cases [44].

Gurm et al [10] also showed that the risk of PC-AKI may vary with the GFR stage for the same CM-dose/GFR ratio [10]. At a g-I/GFR ratio of 0.7–1.0, the incidence increased from less than 2% at GFR ≥ 60 mL/min/1.73 m^2^ to about 4% (dialysis ≈0.25%) at GFR 30–59 mL/min/1.73 m^2^ and to about 15% (requiring dialysis ≈2%) at GFR < 30 mL/min/1.73 m^2^. When the ratio was kept < 0.7, the risk of PC-AKI in patients with GFR < 30 mL/min/1.73 m^2^ decreased to about 6% with no one requiring dialysis.

### CM-dose/GFR ratio faultiness

Studies evaluating the CM dose/GFR ratio suffer from certain principal inaccuracies. Among studies using absolute GFR to express CM-dose/GFR ratio, all have used the CG equation (Table S1). The CG equation was developed in 1976 to estimate endogenous creatinine clearance [45], today a non-acceptable method as a measure of GFR [46]. It was also based on a non-standardized creatinine assay, not compatible with today’s assays traceable to international reference materials [47]. The CG formula has been demonstrated to have substandard accuracy with a systematic overestimation of GFR [41] and may thus result in falsely too-low ratios. Likewise, if the MDRD equation is used, it should be noted that the one containing the 175 coefficient is based on standardized creatinine assays but not the one containing 186. Thus, only equations based on standardized creatinine assays should be used to estimate both relative and absolute GFR, the latter by deindexing relative GFR. The performance of the equation should also have sufficient accuracy in the applied population. It should also be noted that creatinine-based GFR estimating equations may be grossly inaccurate in e.g. patients with abnormal muscle mass or liver failure. In such patients, a cystatin C–based equation or clearance measurements may be preferable [14].

Those applying modern GFR equations based on standardized creatinine assays do not seem to consider the fact that they primarily estimate relative GFR (Table S1). This results in falsely high and low GFR values relative to the true excretion capacity (absolute GFR) in small and large individuals, respectively. Consequently, this will result in falsely low ratios in small individuals and falsely high ratios in large ones, most commonly men in the Western world with an average BSA of about 2.0 m^2^. To calculate the g-I/GFR_ABS_ ratio with modern GFR equations expressing relative GFR, they must first undergo a BSA deindexation (see Eq. 3 above).

A final default of studies evaluating CM dose/GFR ratio is that information about the used creatinine assay and its standardization is not defined, a crucial point with regard to the selected GFR estimating equation.

### Expressing contrast medium dose

Expressing CM dose in terms of gram iodine should be preferred to simply using volume since concentrations of commercially available CM may vary from 140 to 400 mg I/mL. Furthermore, common g-I doses at CT and angiography-based procedures may vary from 15 g-I (= 50 mL × 300 mg I/mL) to 100 g-I (≈300 mL × 320 mg I/mL) and are in the same numerical range as the patients’ GFR_ABS_, i.e., 15–100 mL/min. Thus, forming a g-I/GFR_ABS_ ratio provides the examiner with a simple numerical relationship to predict the risk of PC-AKI as pointed out almost 20 years ago [20].

### Second-pass renal CM exposure examinations

CM-dose/GFR_ABS_ ratio thresholds for increased risk of PC-AKI derived from PCA/PCI studies may also be applied to other procedures where CM reaches the renal arteries after dilution by circulation through the right heart and pulmonary circulation or a systemic capillary bed, s.c. second-pass renal exposure [3]. This includes intra-arterial CM injections into the coronary, carotid, subclavian, mesenteric and iliofemoral arteries, and infrarenal aorta as well as intravenous injections [3, 48].

It may be argued that backflow of CM into the aorta, as well as a left ventriculogram during PCA/PCI, implies that CM may reach the kidneys during its first pass in a relatively undiluted form and with a more toxic effect compared to a pure intravenous injection. However, only about 20% of cardiac output reaches the renal arteries. This means that only a small amount of the CM backflow and, e.g., 6 of 30 mL for a left ventriculogram, will reach the kidneys during its first pass. Thus, first-pass renal CM exposure constitutes only a minor part of a not uncommonly total dose of 200–300 mL given during PCA/PCI [9, 10], probably also fairly diluted when reaching the kidneys. Also note that the injection dose rate may be up to a factor 100 times higher during CT, due to much shorter injection time (typically 30 s) compared with PCA/PCI where repeated small doses may be given over a span of 30–60 min [49], which is another factor to consider regarding toxicity [50]. Unfortunately, there is not a single CT study analyzing the value of the CM-dose/GFR_ABS_ ratio.

### Computed tomography

The incidence of PC-AKI has been shown to be similar for CT and PCA/PCI in four retrospective studies [51–54] and with the patient as its own control in three of them [51–53]. One retrospective study with propensity score matching [55] and one prospective hydration study [56] came up with similar results. In a retrospective study by From et al, a higher risk of PC-AKI-associated 30-day mortality was observed after intravenous injections as compared to intra-arterial injections after adjustment for risk factors [57]. However, in one small single-center randomized study comparing coronary CT-angiography (median 23-g iodine) with PCA (median 27-g iodine including 9.6-g iodine for left heart ventriculography in 86% of the patients) and PCI in 12% of the patients, PC-AKI was significantly more common following PCA, 13.2% vs. 5.6% [58].

Based on these studies and the fact that intravenous CM injections at CT imply an indirect (second pass) renal exposure similar to that from PCA/PCI, the Contrast Media Committee of the Swedish Society of Uroradiology recommends keeping the g-I/GFR_ABS_ ratio < 1.0 also at CT [5]. In patients at risk of CI-AKI (Table 4) [59] the committee recommends aiming at a ratio < 0.5 at CT by reducing the CM dose according to the 10-to-10 rule [60]. This can be achieved by applying a low kilovoltage technique combined with tube loading compensation to prevent an increase in image noise [61–67]. No other organization gives any specific recommendations regarding CM dose in relation to GFR at CT. The Swedish Society recommendations have been implemented in a dose calculator called OmniVis, which has been used in Sweden for twenty years and is also available in Norway, the UK, and the Benelux Union.

**Table 4:** GFR thresholds indicating a risk of contrast medium-induced acute kidney injury following intravenous injections according to various organization

Organization	Risk factors
American College of Radiology [2]	• GFR < 30 mL/min/1.73 m^2^ • In individual high-risk circumstances (eg, numerous risk factors, recent AKI, borderline GFR), prophylaxis may be considered in patients with GFR of 30–44 mL/min/1.73 m^2 ^at the discretion of the ordering clinician
European Society of Urogenital Radiology [3, 4]	• GFR < 30 mL/min/1.73 m^2^
Swedish Society of Uroradiology, revised guidelines 2022 [59]	• eGFR < 30 mL/min/1.73 m^2^ • eGFR 30–44 mL/min/1.73 m^2^ when gram-iodine/GFR_Absolute_ ratio > 0.5 • eGFR 45–59 mL/min/1.73 m^2^, ≥ 2 risk factors* and when gram-iodine/GFR_ABS_ ratio > 0.5, *eg, diabetes, chronic heart failure NYHA III/IV, NSAID or nephrotoxic drugs • eGFR ≥ 60 mL/min/1.73 m^2^ when gram-iodine/GFR_ABS_ ratio > 1.0

*eGFR* estimated glomerular filtration rate, *ICU* intensive care unit, *NSAID* non-steroidal anti-inflammatory drugs*, NYHA* New York Heart Association

### Future CT CI-AKI studies

Today’s controversies regarding the true risk of CI-AKI at CT are to a large extent due to the lack of any randomized controlled studies. Present international guidelines are mainly based on retrospective propensity score-matched controlled studies [2–4], which has been regarded as low-grade evidence [68] with an obvious risk for selection bias between the CM and non-CM groups [69, 70]. It has been suggested that a more appropriate approach would be to restrict the analysis to the CM-enhanced CT group and propensity match patients at various relative GFR intervals (e.g., < 30, 30–44, and 45–59 mL/min/1.73 m^2^) with different CM-dose/GFR_ABS_ ratios [69]. However, this requires documentation of CM doses and concentrations, laboratory-reported relative eGFR, and height and weight to calculate BSA and achieve deindexed absolute GFR. Another option would be to prospectively study patients with malignant diseases undergoing regular surveillance with both non-enhanced and CM-enhanced CT and to perform the two phases with a week apart. Then the patient would also be under its own control.

## eGFR_cystatin C_/eGFR_creatinine_ and glomerular hypofiltration syndromes

Estimation of GFR using both creatinine- and cystatin C–based equations has resulted in an anticipated new set of kidney disorders, selective glomerular hypofiltration syndrome (SGHS) [71] as first suggested by Grubb et al [72]. The syndrome is characterized by a selective reduction in the filtration of medium-sized 5–30-kDa molecules, such as cystatin C (13.3 kDa), compared to the filtration of small molecules < 1 kDa such as creatinine (0.113 kDa). The syndrome has been defined as an eGFR_cystatin C_/eGFR_creatinine_ ratio < 0.6 or 0.7 in the absence of extrarenal influences on cystatin C (e.g., moderate or high doses of glucocorticoids) or creatinine (e.g., low muscle mass). SGHS has been associated with a substantial increase in morbidity and mortality including patients with normal mGFR [73] which might be caused by accumulation of atherosclerosis-promoting proteins [74].

In a recent retrospective study in patients undergoing PCI, multivariate logistic regression analysis indicated that SGHS (eGFR_cystatin C_/eGFR_creatinine_ ratio < 0.6) was significantly associated with AKI and had stronger predictive power for AKI than creatinine‐based eGFR [75]. Notably, SGHS was associated with an increased AKI risk irrespective of whether chronic kidney disease (eGFR_creatinine_ < 60 mL/min/1.73 m^2^) was present or not.

## Take home messages

1. *Relative GFR (mL/min/1.73 m*^*2*^*)* should be used to assess the GFR category as a sign of the degree of kidney damage and sensitivity for CI-AKI.
2. *Absolute GFR (mL/min)* is the individual’s actual excretion capacity. It can be used to calculate an upper limit of the total CM dose in relation to this capacity (i.e., the CM-dose/GFR_ABS_ ratio) to minimize the risk of CI-AKI depending on the degree of kidney damage.
3. *CM-dose/GFR*_*ABS*_* ratio* is directly related to systemic CM exposure and in turn to toxicity. The total dose should be expressed in grams of iodine and not in mL because CM concentrations have a great variation. A gram-I/GFR_ABS_ ratio < 1.0 has been recommended for intra-arterial second-pass renal exposures and may also be adapted for intravenous injections. In patients at risk of CI-AKI, it may be advisable to keep the ratio < 0.5 if possible, without degrading the diagnostic quality. This may be achieved with a low-kilovoltage technique for CT.
4. *Estimation of GFR should* only use equations based on internationally standardized creatinine or cystatin C assays.
5. *Unstable renal function* makes accurate estimation of GFR with creatinine- or cystatin C–based equations impossible.
6. *Prospective studies* are warranted to evaluate the optimal CM-dose/GFR_ABS_ ratio to predict CI-AKI at various stages of kidney damage for CT and intra-arterial examinations based on modern GFR estimating equations.
7. *Selective glomerular hypofiltration syndrome* defined as eGFR_cystatin C_/eGFR_creatinine_ < 0.6 may represent a new and more powerful phenotype of renal dysfunction to predict AKI following CM examinations but needs to be confirmed in further trials.

### Supplementary information

Below is the link to the electronic supplementary material.

Supplementary file1 (PDF 220 kb)

## Abbreviations

ABS: Absolute
AKI: Acute kidney injury
AUC: Area under the plasma concentration–time-curve
BSA: Body surface area
CG: Cockcroft-Gault
CI-AKI: Contrast medium-induced acute kidney injury
CKD-EPI: Chronic Kidney Disease Epidemiology collaboration equation
CM: Contrast media
CT: Computed tomography
eGFR: Estimated glomerular filtration rate
EKFC: European Kidney Function Consortium equation
GFR: Glomerular filtration rate
g-I: Gram iodine
KDIGO: Kidney Disease: Improving Global Outcomes
LMR: Lund-Malmö Revised equation
MDRD: Modification of Diet in Renal Disease equation
mGFR: Measured glomerular filtration rate
PCA: Percutaneous coronary angiography
PC-AKI: Post contrast medium-induced acute kidney injury
PCI: Percutaneous coronary interventions
REL: Relative
SGHS: Selective glomerular hypofiltration syndromes

## Declarations

### Guarantor

The scientific guarantor of this publication is Ulf Nyman.

### Statistics and biometry

No complex statistical methods were necessary for this paper.

### Methodology

• Not applicable
