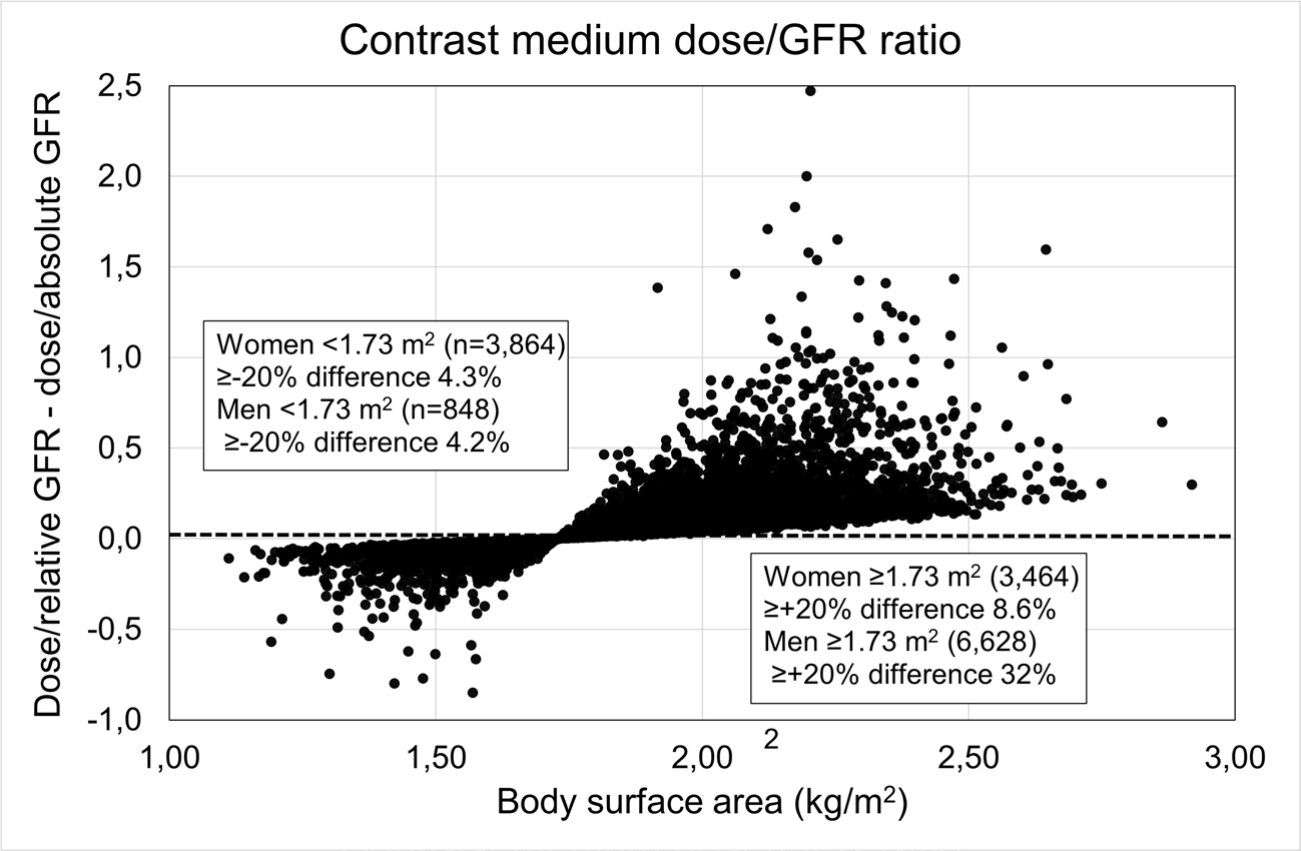# Correction: Absolute and relative GFR and contrast medium dose/GFR ratio: cornerstones when predicting the risk of acute kidney injury

**DOI:** 10.1007/s00330-023-10199-w

**Published:** 2023-09-05

**Authors:** Ulf Nyman, Peter Leander, Per Liss, Gunnar Sterner, Torkel Brismar

**Affiliations:** 1 Department of Translational Medicine, Division of Medical Radiology, University of Lund, Malmö, Sweden; 2 Department of Surgical Sciences, Section of Radiology, Uppsala University, Uppsala, Sweden; 3 Department of Nephrology, Skåne University Hospital, Malmö, Sweden; 4 Division of Medical Imaging and Technology, Department of Clinical Science, Intervention and Technology (CLINTEC), Karolinska Institute/Karolinska University Hospital, Stockholm, Sweden; 5 Department of Radiology, Karolinska University Hospital in Huddinge, Stockholm, Sweden

**Correction: European Radiology**

https://doi.org/10.1007/s00330-023-09962-w

The original version of this article, published on 4 August 2023, unfortunately contained some mistakes. First of all, Fig. 2B has been replaced by an updated version. Additionally, the ratio in the following phrase “it results in a relatively high ratio (1.11) and thus a greater CI-AKI risk” in the caption of Table 2 has been corrected to (1.00). Finally, the following information has been added to the Acknowledgements section: "All authors are members of the Contrast Media Committee of the Swedish Society of Uroradiology (PLe chairman, PLi head of iodine contrast media group),

TB is a member of the Contrast Media Committee of the European Society of Urogenitalradiology

GS member of the Swedish Society of Nephrology

UN is a member of European Kidney Function Consortium, which is endorsed by the European Renal Association". The original article has been corrected.