# Correction to: Absolute and relative GFR and contrast medium dose/GFR ratio: cornerstones when predicting the risk of acute kidney injury

**DOI:** 10.1007/s00330-023-10314-x

**Published:** 2023-10-18

**Authors:** Ulf Nyman, Peter Leander, Per Liss, Gunnar Sterner, Torkel Brismar

**Affiliations:** 1 Department, of Translational Medicine, Division of Medical Radiology, University of Lund, Malmö, Sweden; 2 Department of Surgical Sciences, Section of Radiology, Uppsala University, Uppsala, Sweden; 3 Department of Nephrology, Skåne University Hospital, Malmö, Sweden; 4 Division of Medical Imaging and Technology, Department of Clinical Science, Intervention and Technology (CLINTEC), Karolinska Institute/Karolinska University Hospital, Stockholm, Sweden; 5 Department of Radiology, Karolinska University Hospital in Huddinge, Stockholm, Sweden

**Correction to: European Radiology**

**https://doi.org/10.1007/s00330-023-09962-w**

The author Ulf Nyman's name was given incorrectly as Nyam U in the footer of the Supplementary file.

The original article has been corrected.

