# Supplementary material for: Absolute and relative GFR and contrast medium dose/GFR ratio: cornerstones when predicting the risk of acute kidney injury
Source: Eur Radiol. 2023 Aug 4;34(1):612–21. doi: 10.1007/s00330-023-09962-w (PMC10791854; doi:10.1007/s00330-023-09962-w)
Supplement: Supplementary file 1 — Supplementary file1 (PDF 220 kb) [file 330_2023_9962_MOESM1_ESM.pdf]

Absolute and relative GFR and contrast medium dose/GFR ratio. Corner stone's when predicting the risk of acute kidney injury

Electronic supplementary material

Table S1. Studies evaluating contrast medium dose/GFR ratio as a significant and independent predictor of post contrast medium-induced acute kidney injury following percutaneous coronary angiography or interventions. Studies in upper part based on absolute GFR estimated by the Cockcroft-Gault equation (non-standardized creatinine assays) and lower part based on relative GFR estimated by equations using creatinine assays traceable to international standards except for 186-MDRD.

Table S2. Contrast medium dose/GFR ratio as a significant and independent predictor of post contrast medium-induced acute kidney injury following percutaneous coronary angiography or interventions. Weighted mean value with individual study sizes as weights calculated based on log-transformation of contrast-volume/GFR and gram-iodine/GFR ratio. Studies in upper part based on absolute GFR estimated by the Cockcroft-Gault equation (non-standardized creatinine assays) and lower part based on relative GFR estimated by equations using creatinine assays traceable to international standards except for 186-MDRD.

Eur Radiol (2023) Nyman U, Leander P, Liss P, Sterner G, Brismar T

Table S1.

First author	Country	Year	Indication	Study design	AKI definition*	eGFR equations	Mean eGFR
Laskey (8)	USA	2007	PCI, unselected	Retrospective registry study	>0.5 mg/dL	Abs CG	87±38
Nyman (9)	Sweden	2008	PCI, STEMI	Retrospective	≥44.2 μmol/L	Abs CG	75 ±25
Amiri (28)	Iran	2018	PCA or PCI, elective w/o MetS	Prospective	≥0.5 mg/dL or ≥25%	Abs CG	82 ±25
Amiri (28)	Iran	2018	PCA or PCI, elective w. MetS	Prospective	≥0.5 mg/dL or ≥25%	Abs CG	82 ±26
Barbieri (29)	Italy	2016	PCA or PCI (48%)	Retrospective registry study	≥0.5 mg/dL or ≥25%	Abs CG	71
Khalil (30)	Egypt	2018	PCI, sCr <1,5 mg/dL	Retrospective	≥0.5 mg/dL or ≥25%	Abs CG	95
Liu (31)	China	2015	PCA or PCI, unselected	Prospective	>0.5 mg/dL	Abs CG	72 ±27
Worasuwannarak (32)	Thailand	2010	CC or PCI, elective diabetics	Prospective	≥0.5 mg/dL or ≥25%	Abs CG	61±27
Abe (33)	Japan	2014	PCI, stable angina	Retrospective registry study	≥0.5 mg/dL or ≥25%	Rel Jap MDRD	66±18
Ando (34)	Italy	2014	PCI, STEMI	Prospective	≥0.5 mg/dLor ≥25%	Rel MDRD	91 ±32
Celik (35)	Turkey	2015	PCI, STEMI	Prospective	≥0.3 mg/dL	Rel 175-MDRD	90.7
Kim (36)	Korea	2014	PCI, CKD	Retrospective registry study	≥0.5 mg/d or, ≥25%	Rel 175-MDRD	38.7
Nie (37)	China	2021	PCA or PCI (60%)	Prospective MC	≥0.5 mg/dL	Rel CKD-EPI	NR
Nozue (38)	Japan	2009	PCI, elective stable angina	Retrospective	>0.5 mg/dL, >25%	Rel Jap MDRD	55 ±22
Yoon (39)	Korea	2011	PCI, elective	Prospective	>0.5 mg/dL or >25%	Rel 186-MDRD	62.8

*Serum creatinine rise.

AKI = acute kidney injury, CC = cardiac catheterization, CKD = chronic kidney disease, MC = multicentre, MetS = metabolic syndrome, NR = not reported, PCA = percutaneous coronary angiography, PCI = percutaneous coronary intervention, sCr = serum creatinine, STEMI = ST-elevated myocardial infarction.

eGFR = estimated glomerular filtration rate, Abs =absolute GFR values in mL/min, Jap = Japanese, Rel = relative GFR values in mL/min/1.73 m^2, CG = Cockcroft-Gault, CKD-EPI = Chronic Kidney Disease Epidemiology Collaboration equation, MDRD = Modification of Diet in Renal Disease study equation with coefficient 175 (standardized) and 186 (non-standardized, respectively.

Table S2.

First author	Total number	PC-AKI incidence	CM volume/ GFR ratio	CM conc	Gram I/ GFR ratio	AUC	Sensi-tivity	Speci-ficity	Adjusted odds ratio	PC-AKI <ratio	PC-AKI ≥ratio
Laskey (8)	3179	1.5%	3.7	350*	1.30§	0.690	65%	75%	3.84 (2.01-7.34)	NR	NR
Nyman (9)	391	16.6%	2.9	350	1,00	0.800	NR	NR	NR	3%	25%
Amiri (28)	125	14.4%	2.0#	320	0.63	0.729 (0.649-0.826)	72%	74%	3.92 (1.8-8.52)	6%	32%
Amiri (28)	130	39.2%	1.6#	320	0.51	0.738 (0.649-0.826)	68%	73%	3.92 (1.8-8.52)	22%	63%
Barbieri (29)	2308	12.2%	6.15	350*	2.15§	NR	34%	86%	1.81 (1.19-2.76)	9.7%	25%
Khalil (30)	314	10.8%	2.86	350*	1.00§	0.887	85.30	79.60	25.1 (8.5-74.2)	NR	NR
Liu (31)	3273	2.6%	2.44	370	0.90§	0.780	73%	70%	4.16 (2.45-7.06)	1.0%	6.3%
Worasu-wannarak (32)	248	5.2%	2.6	370*	0.98	NR	NR	NR	5.8 (1.7-19.4)	NR	NR
Subtotal	*9968*		*3.47†*		*1.24†*						
Abe (33)	1222	4.2%	3.0	350	1.05§	NR	NR	NR	2.07 (1.01-4.26)	3.1%	6.1%
Ando (34)	470	5.3%	2.5	385*	0.96§	0.77 (0.66-0.87)	72%	78%	5.6 (1.9-15.4)	NR	NR
Celik (35)	597	13.1%	2.0	350*	0.70§	0.786 (0.729-0.844)	70%	78%	5.9 (2.8-12.3)		
Kim (36)	297	18.5%	6.0	350*	2.10§	0.620	53%	72%	NR	NR	NR
Nie (37)	4254	1.7%	1.78	350*	0.62§	0.736 (0.670-0.803)	61%	75%	2.66 (1.50-4.72)	NR	NR
Nozue (38)	60	13.3%	5.1	370	1.89§	0.839	88%	75%	NR	NR	NR
Yoon (39)	226	7.1%	4.2#	335*	1.42	0.867	81%	80%	9.79 (3.40-28.2)	1.8%	24%
Subtotal	*7126*		*2.19†*		*0.77†*						
Total	*17094*		*2.86†*		*1.02†*						

*Anticipated mean concentration, #Calculated based upon CM concentration and reported gram-iodine/GFR ratio, §Calculated based upon CM concentration and reported CM-volume/GFR ratio, †Weighted mean value.
AUC = Receiver-operating characteristic curve analysis to determine the best cut-off point for CM-dose/GFR ratio to predict PC-AKI with area under the curve (AUC) as a measure of its accuracy. Adjusted odds ratio of CM-dose/GFR ratio as a predictor of PC-AKI.
CM = contrast medium, GFR = glomerular filtration rate, I = iodine, PC-AKI = post contrast medium-induced acute kidney injury.

References

8. Laskey WK, Jenkins C, Selzer F, et al (2007) Volume-to-creatinine clearance ratio: a pharmacokinetically based risk factor for prediction of early creatinine increase after percutaneous coronary intervention. J Am Coll Cardiol 50:584-590

9. Nyman U, Björk J, Aspelin P, Marenzi G (2008) Contrast medium dose-to-GFR ratio: A measure of systemic exposure to predict contrast-induced nephropathy after percutaneous coronary intervention. Acta Radiol 49:658-667

28. Amiri A, Ghanavati R, Riahi Beni H, Sezavar SH, Sheykhvatan M, Arab M (2018) Metabolic Syndrome and the Iodine-Dose/Creatinine Clearance Ratio as Determinants of Contrast-Induced Acute Kidney Injury. Cardiorenal Med 8:217-227

29. Barbieri L, Verdoia M, Marino P, Suryapranata H, De Luca G (2016) Contrast volume to creatinine clearance ratio for the prediction of contrast-induced nephropathy in patients undergoing coronary angiography or percutaneous intervention. Eur J Prev Cardiol 23:931-937

30. Khalil WA, El-Awady W, El-Menshawy MD, Emad M (2018) The early detection and prevention of contrast induced nephropathy post coronary intervention in catheterization unit. J Ind Coll cardiol 8:157-161

31. Liu Y, Chen JY, Tan N, et al (2015) Safe limits of contrast vary with hydration volume for prevention of contrast-induced nephropathy after coronary angiography among patients with a relatively low risk of contrast-induced nephropathy. Circ Cardiovasc Interv 8

32. Worasuwannarak S, Pornratanarangsi S (2010) Prediction of contrast-induced nephropathy in diabetic patients undergoing elective cardiac catheterization or PCI: role of volume-to-creatinine clearance ratio and iodine dose-to-creatinine clearance ratio. J Med Assoc Thai 93 Suppl 1:S29-34

33. Abe D, Sato A, Hoshi T, et al (2014) Clinical predictors of contrast-induced acute kidney injury in patients undergoing emergency versus elective percutaneous coronary intervention. Circ J 78:85-91

34. Ando G, de Gregorio C, Morabito G, Trio O, Saporito F, Oreto G (2014) Renal function-adjusted contrast volume redefines the baseline estimation of contrast-induced acute kidney injury risk in patients undergoing primary percutaneous coronary intervention. Circ Cardiovasc Interv 7:465-472

35. Celik O, Ozturk D, Akin F, et al (2015) Association Between Contrast Media Volume-Glomerular Filtration Rate Ratio and Contrast-Induced Acute Kidney Injury After Primary Percutaneous Coronary Intervention. Angiology 66:519-524

36. Kim JH, Yang JH, Choi SH, et al (2014) Predictors of outcomes of contrast-induced acute kidney injury after percutaneous coronary intervention in patients with chronic kidney disease. Am J Cardiol 114:1830-1835

37. Nie Z, Liu Y, Wang C, Sun G, Chen G, Lu Z (2021) Safe Limits of Contrast Media for Contrast-Induced Nephropathy: A Multicenter Prospective Cohort Study. Front Med (Lausanne) 8:701062

38. Nozue T, Michishita I, Iwaki T, Mizuguchi I, Miura M (2009) Contrast medium volume to estimated glomerular filtration rate ratio as a predictor of contrast-induced nephropathy developing after elective percutaneous coronary intervention. J Cardiol 54:214-220

39. Yoon HJ, Hur SH (2011) Determination of safe contrast media dosage to estimated glomerular filtration rate ratios to avoid contrast-induced nephropathy after elective percutaneous coronary intervention. Korean Circ J 41:265-271
